# Molecular Insights into PKA Activity Modulation by Lysine Acetylation

**DOI:** 10.21203/rs.3.rs-10618766/v1

**Published:** 2026-08-31

**Authors:** Analia G Novero, Iñaki Gentile, Franco A Biglione, Arturo Matamoros Volante, Carla Ritagliati, Tomás Steeman, Mariano G Buffone, Diego Krapf, German L Rosano, Andres Binolfi, Cintia Stival, Dario Krapf

**Affiliations:** Instituto de Biología Molecular y Celular de Rosario; Instituto de Biología Molecular y Celular de Rosario; Instituto de Biología Molecular y Celular de Rosario; Colorado State University; Weill Cornell Medicine; Instituto de Biología Molecular y Celular de Rosario; Experimental Medicine and Biology Institute; Colorado State University; Instituto de Biología Molecular y Celular de Rosario; Instituto de Biología Molecular y Celular de Rosario; Instituto de Biología Molecular y Celular de Rosario; Instituto de Biología Molecular y Celular de Rosario

**Keywords:** PKA, sperm capacitation, NMR, MS/MS, lysine acetylation, fertility, super resolution microscopy

## Abstract

**Background:**

Mammalian sperm capacitation is a critical maturation process regulated by the cAMP-dependent protein kinase A (PKA) signaling axis. While cAMP levels peak early and decline during capacitation, PKA activity remains paradoxically sustained.

**Methods:**

Here, we used a combination of in vitro acetylation assays, enzyme kinetic analysis, nuclear magnetic resonance (NMR) spectroscopy, and mass spectrometry to shed light on this knowledge gap.

**Results:**

We identified lysine acetylation as a novel post-translational modification that modulates PKA activity and localization in mouse sperm. Acetylation of the PKA catalytic subunit (PKA-C) enhances its activity by increasing its binding affinity for ATP, suggesting the existence of cAMP-independent regulatory mechanisms. Furthermore, we show that acetylation promotes the dissociation of the PKA holoenzyme, mimicking the activating effect of cAMP.

**Conclusions:**

These findings uncover a multimodal regulatory mechanism where lysine acetylation acts independent of cAMP to sustain PKA signaling during sperm maturation. Our study provides a molecular basis for the persistent activation of PKA in transcriptionally silent cells and highlights a potentially conserved regulatory principle for this ubiquitous eukaryotic protein kinase family.

## BACKGROUND

After ejaculation, mammalian spermatozoa undergo a complex maturation process termed capacitation, which renders them competent to fertilize the oocyte [1, 2]. Capacitation occurs naturally within the female reproductive tract but can also be induced in vitro by incubating sperm in defined media containing albumin, calcium, bicarbonate, and energy substrates [3]. This process entails extensive biochemical and structural remodeling, including an increase in membrane fluidity, dynamic changes in intracellular ion concentrations (notably Ca^2+^ and HCO_3_^−^), and hyperpolarization of the plasma membrane [4]. At the functional level, capacitation culminates in hyperactivated motility and the ability to undergo the acrosome reaction, both of which are prerequisites for successful fertilization.

At the signaling level, the cAMP–PKA axis represents the central regulatory pathway during capacitation. Bicarbonate and calcium ions activate the soluble adenylyl cyclase ADCY10, producing a rapid rise in intracellular cAMP that in turn activates protein kinase A (PKA) [5, 6] a Ser/Thr kinase also known as cyclic AMP-dependent protein kinase (EC: 2.7.1.37). The structure of PKA was resolved by X-ray diffraction in 1991 and has since become the prototype of the eukaryotic protein kinase family [7]. In its inactive state, PKA consists of two regulatory (PKA-R) and two catalytic (PKA-C) subunits (for a comprehensive review see Baro Graf et al., 2020) [8]. Notably, this holoenzyme composition has been recently challenged, showing that, at least in bovine sperm, it consists of one PKA-R subunit paired with a single PKA-C subunit [9]. Traditional paradigm states that both in somatic and in reproductive cells, cooperative binding of cAMP to the PKA-R subunits releases the active PKA-C subunits, enabling phosphorylation of multiple downstream targets [4]. Phosphorylation by PKA regulates motility, metabolism, proteasome function, and other capacitation-associated processes.

Recent evidence indicates that cAMP is not the only regulator of PKA activity during capacitation. In murine sperm, cAMP levels peak within the first 10 minutes of capacitation before declining, consistent with a transient bicarbonate-induced cAMP response [10]. PKA activity, by contrast, remains elevated well beyond this early cAMP peak, suggesting that additional mechanisms support PKA activity independently of sustained cAMP elevation [10]. In this sense, post-translational modifications (PTMs) play a crucial role in sperm capacitation, as these cells are transcriptionally and translationally silent [11]. Among PTMs, lysine acetylation has emerged as a key regulatory mechanism in both somatic and germ cells [12, 13]. Initially characterized as a histone modification that regulates chromatin accessibility, acetylation is now recognized as a widespread modification affecting thousands of non-histone proteins, including metabolic enzymes, chaperones, transcription factors, and kinases [13–16]. Acetylation alters protein charge, conformation, stability, enzymatic activity, and protein–protein interactions, thereby reshaping entire signaling networks [15–18]. Specifically, acetylation dynamics is controlled by acetyltransferases (KATs) and deacetylases (HDACs), providing a distinct regulatory layer that often integrates with phosphorylation-based signaling.

In the context of sperm, global proteomic analyses revealed an unexpectedly rich acetylome. In capacitated human sperm, acetylation has been mapped to hundreds of proteins involved in flagellar function, energy metabolism, proteasome activity, and signaling pathways [19–21]. PKA-C has been found acetylated in a human acetylome study only under capacitating conditions [19]. Moreover, acetylation has been reported on multiple mitochondrial and glycolytic enzymes in sperm, linking this PTM to energy production and motility, two processes coupled to PKA activity [20, 21].

Our previous work demonstrated that capacitation-related events (*i.e*. sperm hyperactivation and plasma membrane hyperpolarization) can be induced by upregulating lysine acetylation in mouse sperm via treatment with deacetylase inhibitors, under conditions that typically do not promote cAMP synthesis [13]. Consistently, acetylated PKA-C was also identified in an acetylation-enriched proteomic dataset of human sperm peptides [19]. These observations suggest that acetylation might promote or sustain PKA activity in a cAMP-independent manner. Acetylation can influence the conformation and dynamics of kinase activation segments, alter ATP-binding affinity, and affect the stability and assembly of kinase holoenzymes. Through these effects, acetylation provides an additional layer of control over kinase signaling, complementing canonical regulatory mechanisms such as phosphorylation and allosteric interactions [22, 23]. In particular, acetylation of kinase catalytic subunits has been described as a mechanism to fine-tune substrate specificity and subcellular localization, highlighting a potentially conserved regulatory principle [24]. Thus, lysine acetylation emerges as a candidate for explaining the persistent activation of PKA during sperm capacitation, despite declining cAMP levels. Yet, the consequences of direct PKA acetylation in sperm remain unexplored.

In this study, we established an *in vitro* system using the recombinant mouse PKA catalytic subunit (rPKA-C) subjected to lysine acetylation by endogenous sperm acetyltransferases, to dissect the molecular impact of this PTM on PKA enzymatic activity. This mouse ortholog was the one used to define the kinase fold for the entire field: the prototypical PKA-C crystal structure [7] was solved using mouse PKA-C (P05132), and it continues to be used interchangeably as a structural reference for PKA more broadly. We further explored the effect of lysine acetylation on PKA regulation in mouse sperm cells during capacitation. We demonstrate that acetylation regulates PKA activity through two complementary mechanisms: *(1)* enhancing catalytic efficiency by increasing ATP binding affinity, and *(2)* promoting dissociation of regulatory and catalytic subunits, mimicking cAMP-induced activation. Collectively, our findings uncover acetylation as a novel and physiologically relevant regulatory layer of PKA activity, opening new perspectives on kinase regulation in both sperm physiology and broader cellular contexts.

## METHODS

### Data, code, and materials availability

Mass spectrometry data were deposited in the ProteomeXchange Consortium via the PRIDE partner repository with the dataset identifier PXD081400.

#### Chemicals and reagents.

The following commercial antibodies were used: rabbit recombinant anti-PKA-RIIα (MAB8000; R&D Biosystems, Minneapolis, MN, USA; [25]), mouse monoclonal anti-PKA-C (clone 5B, 610980; BD Transduction Laboratories, Franklin Lakes, NJ, USA; [26], horseradish peroxidase (HRP)-conjugated anti-mouse IgG (7076) and anti-rabbit IgG (7074; Cell Signaling Technology, Danvers, MA, USA), anti-acetyl-lysine antibody (9441S; rabbit polyclonal; Cell Signaling Technology) and anti-phospho-PKAc (T197) antibody (5661; rabbit monoclonal; Cell Signaling Technology). Anti-β-tubulin (clone E7) was purchased from the Developmental Studies Hybridoma Bank. Secondary antibodies included Alexa Fluor Plus 488-conjugated anti-mouse IgG (A32723), Alexa Fluor Plus 568-conjugated anti-mouse IgG (A11004), and Alexa Fluor 647-conjugated anti-rabbit IgG (A20991), all from Thermo Fisher Scientific (Waltham, MA, USA). Unless otherwise indicated, bovine serum albumin (BSA), poly-L-lysine, and all other reagents were purchased from Sigma-Aldrich (St. Louis, MO, USA).

#### Mouse Sperm Preparation.

Experiments involving animals were conducted in accordance with the Guide for Care and Use of Laboratory Animals published by the NIH. The study protocols were approved by the Animal Care and Use Committee of the Facultad de Ciencias Bioquímicas y Farmacéuticas de Rosario, UNR (ref 492/2018) and the Animal Care and Use Committee (IACUC) guidelines of Colorado State University (protocol #4759). Cauda epididymal mouse sperm were collected from C57BL/6 young adult male mice (8–13 weeks old) or CD-1 male mice (12–19 weeks old and was placed in 500 μl of a modified Krebs-Ringer solution H-TYH HEPES-buffered medium containing 119.3 mM NaCl, 4.7 mM KCl, 1.2 mM KH_2_PO_4_, 1.2 mM MgSO_4_, 5.6 mM glucose, 0.5 mM sodium pyruvate, 1.7 mM Ca^2+^, and 20 mM HEPES (pH 7.4). The H-TYH medium accounts for non-capacitating medium. After 20 min, epididymides were removed, and the suspension was adjusted with non-capacitating medium to a final concentration of 1–2×10^7^ cells/ml. For capacitation, BSA and NaHCO_3_ were added to final concentrations of 5 mg/ml and 15 mM, respectively.

#### Expression and purification of recombinant PKA catalytic subunit.

The plasmid containing the coding sequence for the recombinant wild-type murine PKA-Cα1 subunit (rPKA-C) was obtained from Addgene (plasmid #14921), which encodes rPKA-C fused to 6xHis-tag [27]. rPKA-C expression and purification were conducted based on established protocols [28, 29], with minor changes. Briefly, a 20 mL starter Luria-Bertani broth culture with 100 μg/mL ampicillin was inoculated with transformed *Escherichia coli* BL21 and allowed to grow overnight at 37°C under constant shaking at 250 rpm. The starter culture was transferred to 1 L of M9 medium (33.7 mM Na_2_HPO_4_, 22 mM KH_2_PO_4_, 8.55 mM NaCl, 1 mM MgSO_4_, 0.1 mM CaCl_2_) supplemented with 1g/L of NH_4_Cl and 4g/L of D-glucose and cells were grown at 37°C until an OD_600_ of 0.4 was reached. Afterward, the temperature was reduced to 18°C and rPKA-C overexpression was induced by the addition of 0.5 mM isopropyl-β-D-thiogalactopyranoside (IPTG, Promega). Expression was allowed to proceed for 12 h with constant shaking at 250 rpm. Cell pellets were harvested by centrifugation at 7,000 g for 20 min at 4°C and resuspended in 20 mL of cold Lysis Buffer (20 mM Tris-HCl pH 8.0, 50 mM KH_2_PO_4_, 100 mM NaCl, 5 mM β-mercapthoethanol, 1 mM PMSF) before sonication for 10 min. Sonication was done on ice with a Branson sonicator device, using 7 pulses of 20 seconds at 35 % power an waiting periods of 2 min periods between pulses to avoid heating. Cell debris was cleared by centrifugation at 9,500 g for 30 min at 4 °C and rPKA-C fused to His-tag was isolated from the supernatants with His-affinity Dynabeads^™^ (Ni-NTA resin). Bound proteins were eluted over a 0–250 mM imidazole gradient dissolved in Lysis Buffer. Fractions containing purified enzyme were pooled. Imidazole was eliminated from the samples, and fractions were concentrated using a 10 kDa MWCO filter Pierce Protein Concentrators (Fisher Scientific). For samples uniformly enriched with ^15^N, rPKA-C (^15^N-rPKA-C) was expressed and purified as described above using M9 minimal medium supplemented with 1 g/L of ^15^NH_4_Cl (Cortecnet, France) and 4 g/L of D-glucose (Sigma). Purity of protein samples was determined by SDS-PAGE analysis and Western-blot using anti-PKA-C antibody (clone 5B, 610980, BD Transduction Laboratories). The recombinant enzyme was expressed at high levels, in both a soluble and active form, as shown later by kinase reaction assays. Noteworthy, the apparent molecular weight of the protein is higher (43 kDa) than reported for endogenous PKA (38 kDa) when analyzed on electrophoresis and Western-blot experiments due to the inclusion of the 6xHis-tag and thrombin cleavage site.

#### In vitro Acetylation of rPKA-C.

To perform the acetylation assays, we used mouse total sperm extracts containing endogenous acetyl transferases. Sperm cells in non-capacitating medium were centrifuged [5,000*g*, 5 min at room temperature (RT)] and resuspended in ice-cold Lysis Buffer (50 mM Tris-HCl, 150 mM NaCl, 1 X EDTA-free protease inhibitor mixture, 0.5% Triton X-100, pH 8), so that the final concentration would be equivalent to 300 ×10^6^ sperm/mL. After 60 min incubation on ice with periodic up-and-down pipetting, sperm extracts were pre-cleared by incubation with Ni^2+^-beads (30 min, 4°C, constant rotation) to eliminate nonspecific binding of sperm proteins. The Ni^2+^-beads volume used for preclearing was half the volume of sperm extract. Then, the beads were pelleted by centrifugation (100 *g*, 1 min, RT), and the supernatant (containing endogenous acetyltransferases) was transferred to a new tube and saved on ice for *in vitro* acetylation assays. This fraction represents the “sperm extract”. Acetylation reactions were performed in a final volume of 60 μL containing 1.5 μg of rPKA-C, sperm extract equivalent to 6×10^6^ cells, 50 mM Tris-HCl, pH 8.0, 1 mM DTT, 150 mM NaCl, 1X EDTA-free protease inhibitor cocktail, 1X PhosSTOP (phosphatase inhibitor cocktail, Sigma), 100 μM of Acetyl-CoA (CoALA Biosciences), and a mixture of deacetylase inhibitors (iDACs, 10 μM TSA and 500 μM NAM). The combination of NAM (nicotinamide) and TSA (Trichostatin A) is commonly used as a broad-spectrum deacetylase inhibitor cocktail, targeting both NAD^+^-dependent sirtuins (class III) and Zn^2+^-dependent histone deacetylases (classes I, II, and IV), thereby effectively inhibiting all major classes of lysine deacetylases [30]. The same reaction buffer without iDACs and Acetyl-CoA was used for control conditions. Samples were incubated at 30°C for 30 min to allow acetylation. Then, rPKA-C was recovered from the reaction mixture by incubation with Ni^2+^-beads for 30 min at RT. Beads were washed twice by centrifugation (100 g, 1 min, 4°C) with 500 μL of PBS. The resulting pellet was treated with 60 μL of 1 M imidazole for 30 min at 4°C to elute rPKA-C. Successful acetylation was verified by Western blot analysis using an acetyl-lysine-specific antibody and mass spectrometric analysis, as explained below.

#### SDS-PAGE and Western blot analysis.

Protein extracts equivalent to 0.5–1×10^6^ sperm/lane were subjected to SDS-PAGE and electrotransferred to PVDF membranes (Bio-Rad) at 250 mA for 90 min at 4°C. Membranes were blocked with 3% BSA in TBS containing 0.1% Tween-20 (T-TBS). Antibodies were diluted in 0.2% Tween 20 T-TBS containing 1% BSA as follows: 1/3,000 for anti-pPKA substrates and 1/10,000 for β-Tubulin. Secondary antibodies were diluted 1/10,000 and 1/20,000, respectively, in 1% fat-free milk - T-TBS and developed using an enhanced chemiluminescence detection kit (ECL KALLIUM Biolumina) according to the manufacturer's instructions. When necessary, PVDF membranes were stripped at 60°C for 15 min in 2% SDS, 0.74% β-mercaptoethanol, and 62.5 mM Tris (pH 6.5) and washed twice for 10 min each time in T-TBS.

#### PKA activity assays and PKA kinetic parameters.

Enzymatic PKA activity was measured using the Kemptide Mobility Shift Assay (KiMSA), as previously described [31, 32]. Briefly, reactions were carried out with 2.5 μL of 0.4 μg/μL Kemptide-FITC (30 μM), 2.5 μL of 0.24 μg/μL non-fluorescent Kemptide (30 μM), and 0.2 μM rPKA-C, under either acetylating or non-acetylating conditions, in a final volume of 25 μL of kinase buffer (final concentrations of 200 mM Tris-HCl pH 7.4, 10 mM MgCl_2_, 0.2 mM ATP, 10 mM DTT, 1X cOmplete EDTA-free protease inhibitor cocktail, and PhosSTOP). Reactions were assembled on ice, incubated for 25 min at 37°C in the dark, and terminated by transferring samples to ice, followed by heating at 95°C for 1 min and addition of 10× loading buffer (63% glycerol, 1% bromophenol blue, 5% Tween-20, 5 mM DTT). Samples were centrifuged at 10,000 × g for 1 min at 4°C, and supernatants were stored on ice or at − 80°C until analysis by agarose electrophoresis.

For determination of kinetic parameters for the Kemptide substrate, kinase reactions contained 3 nM rPKA-C (either previously acetylated or not), 10 mM MgCl_2,_ 50 mM DTT, Protease inhibitor EDTA-free cocktail (Roche) and PhosSTOP in 50 mM Tris buffer pH 7.4. For this set of experiments, a fixed concentration of 200 μM ATP was used, while the concentration of the peptidic substrate was either 5, 16, 20 and 30 μM (maintaining a 1:1 proportion of non-FITC-labeled and FITC-labeled Kemptide).

When assaying the kinetic parameters for ATP, the total Kemptide concentration was kept constant at 200 μM (150 μM non-labeled Kemptide + 50 μM FITC-labeled Kemptide), while changing ATP concentrations between 15, 40, 100 and 200 μM. For determination of kinetics parameters for ATP, kinase reactions contained 8 nM rPKA-C (either previously acetylated or not), 10 mM MgCl_2_, 50 mM DTT, protease inhibitor EDTA-free cocktail (Roche) and PhosSTOP in 50 mM Tris buffer pH = 7.4. In all cases, assays were conducted at 30°C in a total reaction volume of 25 μL, with varying incubation times (0, 5, 10, 15, and 25 min). Negative controls omitted rPKA-C. Following densitometry analysis, the amount of phosphorylation was determined for each Kemptide or ATP concentration by integrating the area of the peaks for the substrate and product. The fraction of phosphorylation was converted to initial reaction velocities (*V*_0_ in μM/min) and plotted against each substrate concentration. Under these kinase reaction conditions, product conversions were ≤ 1% at substrate concentrations equal to Michaelis constant *K*_*m*_, ensuring that the experiments were run under initial velocity conditions. Curve fitting and the determination of kinetics parameters (maximum velocity *V*max, *K*_m_ and catalytic constant *K*_cat_) were performed in GraphPad Prism 8.0.1 (GraphPad, La Jolla, CA, USA) using nonlinear regression to the Michaelis–Menten equation *V*_0_ = *V*_max_ [*S*]/(*K*_m_ + [*S*]), where [*S*] is the substrate concentration and *K*_cat_ is the maximum velocity *V*_max_ normalized to the total enzyme concentration. Three experimental replicates were carried out for each curve, and the mean and SEM for each kinetic parameter were determined.

### Liquid chromatography - mass spectrometry/mass spectrometry (LC-MS/MS) analysis

Sperm protein extracts supplemented with rPKA-C and incubated under either acetylating or non-acetylating conditions, as described in the section “In Vitro Acetylation of rPKA-C,” were subjected to SDS-PAGE. Electrophoresis was stopped after proteins migrated approximately 1 cm into the resolving gel. Gels were stained with colloidal Coomassie, and the protein-containing regions were excised and subjected to in-gel tryptic digestion according to the protocol described by Link and LaBaer to generate peptides for subsequent analysis [33].

Peptide separations were carried out on a nanoHPLC Ultimate3000 (Thermo Scientific) using a nano column EASY-Spray ES901 (15 cm × 50 μm ID, PepMap RSLC C18). The mobile phase flow rate was 300 nL/min using 0.1% formic acid in water (solvent A) and 0.1% formic acid and 100% acetonitrile (solvent B). The gradient profile was 4–30% solvent B for 64 min, 30%-80% solvent B for 7 min, and 90% solvent B for 1 min.

MS analysis was performed using a Q-Exactive HF mass spectrometer (Thermo Scientific). For ionization, 1,9 kV of liquid junction voltage and 250°C of capillary temperature were used. The full scan method employed a m/z 220–3100 mass selection, an Orbitrap resolution of 120,000 (at m/z 200), a target automatic gain control (AGC) value of 10^6,^ and a maximum injection time of 100 ms. After the survey scan, the 20 most intense precursor ions were selected for tandem mass spectrometry (MS/MS) fragmentation. Fragmentation was performed with a normalized collision energy of 28 eV, and MS/MS scans were acquired with a dynamic first mass. The AGC target was 5×10^5^, resolution of 30,000 (at m/z 200), intensity threshold of 1.5×10^5^, isolation window of 1.4 m/z units, and maximum injection time of 55 ms. Charge state screening was enabled to reject unassigned, singly charged, and equal to or more than six protonated ions. A dynamic exclusion time of 60 s was used to discriminate against previously selected ions.

MS data were analyzed with ProteomeDiscoverer 2.4 using standardized workflows. Mass spectra *.raw files were searched against the Uniprot database from *Mus musculus* (reviewed and unreviewed proteins, entry UP000000589) supplemented with rPKA-C sequence. Precursor and fragment mass tolerance were set to 10 ppm and 0.02 Da, respectively, allowing 2 missed cleavages. Carbamidomethylation of cysteines and ^15^N labeling on nitrogen atoms were set as fixed modifications. Variable modifications were protein N-terminal acetylation and methionine loss, methionine oxidation, and lysine acetylation [+ 42.011 Da]. Mass spectrometry data were deposited in the ProteomeXchange Consortium via the PRIDE partner repository with the dataset identifier PXD081400 [34].

### Nuclear magnetic resonance (NMR) spectroscopy

All NMR acquisitions were done at 27 °C on a 700 MHz Bruker Avance III equipped with a TXI (5 mm ^1^H/D-^13^C/^15^N) probe. Spectra of ^15^N-uniformly enriched r-PKA-C in the absence or presence of 2 mM ATP were done on 200 μM samples dissolved in NMR buffer (20 mM KH_2_PO_4_ pH 6.5, 90 mM KCl, 10 mM MgCl_2_) supplemented with 10% D_2_O (99.9% Sigma). For sperm extract samples, we spiked 200 μM ^15^N-rPKA-C into 200 μL control and acetylating extracts supplemented with 25 μL of D_2_O and incubated the mixtures for 2 h before transferring them to Shigemi NMR tubes (Shigemi, Japan) and starting acquisitions. In all cases we used a T1 relaxation optimized version of the HSQC pulse sequence [35]. NMR spectra were acquired with 1K and 128 points, in the ^1^H and ^15^N dimension respectively. We used 1K scans, a recycling delay of 0.14 s and sweep widths of 16 and 36 p.p.m in ^1^H and ^15^N (offsets 4.7 and 119 p.p.m.). Processing was done by zerofilling the FIDs to 4K and 2K in ^1^H and ^15^N dimensions, multiplication by a qsine window function, Fourier transform and baseline correction in both dimensions. Spectra were acquired and processed with Topspin 3.7 (TopSpin, Bruker BioSpin GmbH, Rheinstetten, Germany). HSQC spectra were analyzed with CCPNMR [36] and peak assignment was transferred from previous works [37]. Quantification and mapping of perturbed residues were performed by calculation of chemical shift perturbations of each residue using the equation $CSP=\sqrt{{\left(\Delta \delta_{H}\right)}^{2}+{\left(0.142*\Delta \delta_{N}\right)}^{2}}$. Mean CSP plus one standard deviation was used as a threshold for identifying perturbed residues under acetylating conditions. All illustrations containing NMR spectra were generated using Python's standard visualization libraries and the nmrglue module (Helmus & Jaroniec, 2013).

### Sperm cAMP quantification

cAMP production of sperm incubated in the presence of 500 μM 3-Isobutyl-1-methylxanthine (IBMX) was measured. Aliquots of 2–4 × 10^6^ cells were incubated in each condition tested. After the appropriate time, sperm were sedimented by centrifugation at 2,000*g* for 3 min and lysed in 200 μL HCl for 10 min. Sperm lysates were centrifuged at 2,000*g* for 3 min, and the cAMP in the supernatant was acetylated and quantified using the Direct cAMP ELISA kit (ENZO, catalog no. ADI-901–066A) according to the manufacturer's instructions.

### Sample preparation for immunostaining

Sperm samples were prepared as described above. Capacitated or non-capacitated sperm incubated for 60 min with or without iDACs were washed and placed on 0.001% poly-L-lysine-coated 22×22 mm glass coverslips at 37°C for 10 min until semi-dried. Attached sperm were fixed with 4% w/v PFA in PBS for 10 min at room temperature, followed by three PBS washes of 5 min each. Cells were permeabilized with 0.5% v/v Triton X-100 in PBS for 5 min at RT and washed thrice with PBS. Cells were incubated with 3% w/v BSA and 10% normal goat serum (NGS) in PBS for 60 min at RT to block non-specific binding sites. Blocking solution was removed and replaced with primary antibody solutions (both anti-PKA-RII and anti-PKA-C at 1:100) in 1% BSA, 10% NGS, and 0.1% Tween-20 in PBS (PBS-T). Incubations were performed overnight at 4°C in a humidified chamber to prevent desiccation. Primary antibodies were washed three times with 0.1% PBS-T. Alexa Fluor Plus-conjugated anti-mouse (488) and anti-rabbit (647) secondary antibodies were diluted 1:500 in a solution containing 1% BSA, 10% NGS, and 0.1% PBS-T. Finally, cells were washed three times with PBS-T and stored in this buffer at 4°C in a humidified chamber until used. Immediately before image acquisition, coverslips were mounted with 10 μl of 1% β-mercaptoethanol buffer and sealed with clear nail polish to prevent oxygen exchange.

### Laser scanning microscopy (LSM) imaging

A LSM 900 Zeiss microscope equipped with an Airyscan 2 module and a 63X 1.4 NA objective (C Plan-Apochromat) was used. Images were acquired using the Zen Black 3.8 software (Zeiss, Oberkochen, Germany). Lasers with 640 and 488 nm wavelengths were used for excitation of Alexa Fluor 647 and 488, respectively, set between 1 and 2% power intensity, and master gain adjusted between 800 and 850 V. For Z-stack acquisition, a distance between planes of 100 nm was chosen. The beginning and end of the Z-stack were manually selected. The total Z range depended on the number and size of the cells in the field of view and varied from 4 to 6 μm, which resulted in 40 to 60 images per stack.

### Image processing and analysis

After acquisition, images were subjected to the Zeiss Joint Deconvolution module to enhance resolution and processed using ImageJ/FIJI. A region of interest was selected in the principal piece, and a profile of at least 10 μm in length was drawn to quantify the localization of the proteins of interest. To report the spread of labeled proteins, the fluorescence of a single peak was fit to a Gaussian function and the full width at half maximum (FWHM) was computed from the line profile. In cases where the profile exhibited two peaks, the fit was performed to the sum of two Gaussian functions with centers xc_1_ and xc_2_ and FWHMs w_1_ and w_2_. Total FWHM in two-peak cases was reported as FWHM = |xc_1_ - xc_2_| + (w_1_ + w_2_)/2.

#### Statistical analysis

Data are expressed as mean ± standard deviation (SD) of at least 3 independent experiments from different mice for all determinations, unless otherwise specified. Statistical analyses and data presentation were performed using GraphPad Prism 8.0.1 (GraphPad, La Jolla, CA, USA). When multiple groups of treatments were compared, one-way ANOVA was used, followed by Tukey’s test when data met the assumption of normality. *P* values are specified for each experiment.

## RESULTS

### Endogenous sperm lysine-acetyltransferases acetylate recombinant PKA catalytic subunit.

To characterize the acetylation of PKA-C, a recombinant His-tagged PKA-Cα1 subunit (rPKA-C) was produced and incubated with whole mouse sperm extracts. These extracts served as a source of endogenous acetyltransferases, under two different conditions: (1) in the presence of acetyl-CoA and a cocktail of deacetylase inhibitors (iDACs), containing 10 μM TSA and 500 μM NAM, to promote lysine acetylation, and (2) without these additions. After incubation, rPKA-C was purified by affinity chromatography using immobilized-Ni^2+^ resin, ensuring selective recovery of the recombinant protein and avoiding contamination from endogenous acetylated sperm proteins. Acetylation was then assessed by Western blot using anti-acetyl-lysine antibodies. As shown in Fig. 1A and B, rPKA-C acetylation was detected following incubation with sperm extracts. Although sperm extract alone was sufficient to induce acetylation, the addition of acetyl-CoA and iDACs substantially increased the extent of acetylation.

Mass spectrometry analysis identified five acetylated lysine residues in rPKA-C after in vitro acetylation using sperm extracts. Residues Lys83 and Lys266 were found acetylated upon incubation with sperm extracts, irrespective of the presence of acetyl-CoA and iDACs, consistent with a faint immunoreactive band detected in the Western blot (Fig. 1A). In contrast, Lys168, Lys279, and Lys309 were acetylated only under conditions that favored acetylation (presence of acetyl-CoA and iDACs). Figure 1C shows these three acetylated lysine residues in the structural context of the protein. Notably, Lys168 is located within the catalytic loop of PKA, where it interacts with the γ-phosphate of ATP and helps stabilize the geometry required for efficient phosphoryl transfer during catalysis [39]. As an example, the mass spectrum shift induced by acetylation (+ 42 Da) of Lys279 is shown in Fig. 1D, mirrored to the mass spectrum of the same peptide in nonacetylated rPKA-C. Spectra of peptides containing Lys168 and Lys309 are shown in Suppl. Figure 1.

Collectively, these results demonstrate that rPKA-C is acetylated by endogenous sperm acetyltransferases, a system to be used as an in vitro model to investigate the functional significance of PKA-C acetylation.

#### Acetylated rPKA-C displays increased catalytic activity

The previously identified acetylated Lys168 is located within a key catalytic region, suggesting that lysine acetylation modulates enzymatic activity. To test this hypothesis, we compared the catalytic activity of rPKA-C with its in vitro acetylated counterpart using the KiMSA enzymatic assay [31, 32]. Briefly, the assay relies on a fluorescent peptide substrate (Kemptide-FITC) that acquires two additional negative charges upon phosphorylation by PKA. The phosphorylated and non-phosphorylated species are then separated by agarose gel electrophoresis based on their distinct migration patterns and subsequently quantified by FITC fluorescence densitometry. Acetylated rPKA-C exhibited higher activity than its non-acetylated counterpart, as evidenced by increased levels of phosphorylated Kemptide.

Representative KiMSA assays performed at different time points are shown in Figs. 2A and B, where each lane corresponds to a distinct time point, and a greater accumulation of phosphorylated Kemptide is observed when acetylated rPKA-C was used. Control experiments using rPKA-C not exposed to sperm extract or sperm extract alone (in the absence of rPKA-C) were performed to assess basal activity of the purified enzyme and to exclude contaminating kinase activity from sperm extracts following Ni^2+^-beads recovery, respectively (Suppl. Figure 2A). A comparison between the activity of rPKA-C and the acetylated counterpart upon a 5 min reaction period is shown in Fig. 2B, revealing a significant increase in catalytic activity of acetylated rPKA-C. These data further substantiate that lysine acetylation enhances the catalytic function of rPKA-C. Equal protein amounts of rPKA-C used in the assays shown in Fig. 2B were confirmed by Western blots (Suppl. Figure 2B).

It has been previously stated that phosphorylation of Thr197 within the activation loop of PKA-C is required for enzyme maturation and maximal catalytic activity [40]. We therefore investigated whether acetylation influences the phosphorylation state of this residue. Mass spectrometry analysis revealed no significant differences: Thr197 was phosphorylated in 93% of tryptic peptides from native rPKA-C compared to 95% of peptides from acetylated rPKA-C. To complement these findings, we performed in vivo experiments in which mouse sperm were incubated with iDACs for 60 min to avoid protein deacetylation. As previously reported, this treatment increased phosphorylation of PKA substrates, consistent with enhanced PKA activity in sperm cells [13]. Western blot analysis using anti-pThr197 antibodies showed no differences in the phosphorylation status of Thr197 between acetylating and non-acetylating conditions (Figs. 2C and D). Notably, capacitation was associated with increased Thr197 phosphorylation of sperm PKA-C (Figs. 2C and D). These results indicate that phosphorylation of Thr197 does not account for the increased catalytic activity of PKA-C observed upon acetylation.

#### Lysine acetylation increases rPKA-C binding affinity for ATP

To investigate the molecular basis underlying the enhanced activity of acetylated rPKA-C, we performed solution-state NMR experiments. ^1^H-^15^N HSQC spectra were acquired from ^15^N-labeled rPKA-C incubated in sperm extracts under either acetylating or non-acetylating conditions. The spectra exhibited broad and overlapped signals due to the high molecular weight of r-PKA-C and the more viscous sperm extract solutions (Fig. 3A), preventing direct observation of lysine ε-amide conversion into acetylated moieties [41]. Nevertheless, clear site-selective chemical shift perturbations and line broadening were detected in the acetylating extract, particularly within the glycine-rich loop spanning Leu40 to Glu64 (Figs. 3A–I), including Ile46, Leu49, Gly50, Gly52, Gly55, Met58, Leu59, and Val60. These chemical shift perturbations were also visible within the activation segment spanning Asp184 to Glu206 (Fig. 3A), including Ala188, Thr197, Glu203, and Ala206, also essential for ATP binding and catalysis. These effects were similar to those obtained when ATP was titrated onto pure ^15^N-rPKA-C (Suppl. Figure 3). Considering that we spiked ^15^N-rPKA-C on identical extract samples, with and without acetyl-CoA and IDACs, we hypothesize that lysine acetylation may influence ATP binding and/or catalytic turnover. To directly test catalytic activities of acetylated and non-acetylated rPKA-C, KiMSA assays were performed across variable substrate concentrations.

Kinetic analyses revealed that lysine acetylation had no significant effect on the kinetic parameters for the peptide substrate Kemptide: V_max_ values were comparable (48.8 μM/min vs. 46.2 μM/min) and Michaelis–Menten constants were nearly identical (K_m_,Kemptide = 27.98 μM vs. 25.08 μM; Figs. 4A and B). In contrast, acetylation markedly improved ATP affinity. The K_m_, ATP decreased by more than one order of magnitude (29.22 μM in the non-acetylated vs. 2.76 μM in the acetylated form, Figs. 4C and D, and Table I), with minimal changes in V_max_ (6.97 vs. 5.97 μM/min; Fig. 4B). These findings align with the NMR data and demonstrate that acetylation selectively enhances ATP binding, thereby increasing rPKA-C catalytic efficiency.

#### Acetylation induces in vivo relocation of PKA-C, similar to the effect of cAMP

Post-translational modifications could regulate the interaction between catalytic and regulatory PKA subunits by altering electrostatic interactions, steric constraints, or conformational dynamics at their interface. Lysine acetylation can neutralize positive charges, increase steric bulk that clashes with tightly packed interfaces, or affect secondary structures, altering the shape of binding surfaces.

Decreased affinity between PKA catalytic and regulatory subunits would lead to enhanced activity, even in the absence of cAMP. We have recently shown that capacitation triggers the relocation of PKA-C and PKA-RII subunits, leading to their separation, with PKA-RII repositioning from its original central location in the axoneme of the flagellum to the CatSper channel signalosome in the plasma membrane [25]. In view of the molecular and biological effects of acetylation on PKA-C, we investigated the effect of lysine acetylation on the spatial dynamics of PKA subunits in sperm cells incubated in non-capacitating conditions supplemented with iDACs and compared to control non-capacitating and capacitating conditions. To this end, we used laser-scanning confocal microscopy with an Airyscan super-resolution module.

Figures 5A–C shows representative two-color images of PKA-RII and PKA-C in mouse sperm. As previously shown, in non-capacitated sperm, both PKA-RII and PKA-C localize within an elongated structure surrounding the axoneme (Fig. 5A). A fluorescence intensity profile along the center of the flagellum reveals a central peak for both subunits, characteristic of a rod-like structure, where PKA-RII and PKA-C remained associated, consistent with previous results [25]. Upon capacitation, the regulatory subunit relocated towards the periphery, losing colocalization with PKA-C in 56% of analyzed cells (n = 16). As expected, PKA-C remained in a central elongated structure, albeit slightly expanded [25, 26]. Fluorescence intensity profiles further supported this differential relocalization of both subunits (Fig. 5A–C right panels). Remarkably, when sperm were incubated under non-capacitating conditions in the presence of iDACs, a capacitation-like redistribution of PKA subunits was observed (Fig. 5C). The resulting pattern mirrored PKA behavior of capacitated cells, suggesting that acetylation plays a role in modulating PKA subunit interactions.

To quantify localization changes of PKA subunits, we analyzed fluorescence intensity profiles and calculated the full-width at half-maximum (FWHM) for each condition. The resulting values are presented in Fig. 6A as box plots for the three experimental conditions: capacitating medium, non-capacitating medium, and non-capacitating + iDACs. A strong correlation was observed between FWHM of PKA-C and PKA-RII in the basal non-capacitated state. Both subunits displayed similar widths in all cells (181 ± 48 nm and 190 ± 42 nm, respectively, n = 12 cells), indicative of the PKA inactive state were the complex PKA-C:PKA-R is observed, as previously shown [25] (Fig. 6B). Consistent with the heterogeneous nature of the sperm population, not all cells exhibited PKA-RII relocalization in response to either capacitating stimulus or iDACs. After a 60 min incubation in capacitating medium, PKA-RII showed a broad distribution range, from 180 to 790 nm (n = 16 cells), highlighting variability in the cellular response. Upon incubation in acetylating conditions, only a subset of cells displayed PKA-RII redistribution with an increased diameter, similar to the response observed upon capacitation. In cells where the PKA-RII diameter remained below 400 nm (7 out of 16 cells), the strong correlation with PKA-C persisted, suggesting that the subunits remained associated. However, when the PKA-RII diameter exceeded 400 nm, this correlation was lost, indicating PKA complex dissociation in both conditions. These quantitative analyses provide compelling evidence showing that relocation of PKA-RII upon sperm stimulation with either capacitating or iDACs-containing media is of a similar extent.

Supporting the hypothesis that a PTM reducing the affinity between PKA-C and PKA-R can result in dissociation of PKA subunits through a cAMP-independent mechanism, incubation of sperm cells with iDACs to promote acetylation did not increase cAMP levels (Fig. 7) at any of the time points evaluated (1 min, 5 min and 15 min). In agreement with this observation, we previously showed that incubation of mouse sperm under non-capacitating conditions with iDACs increases phosphorylation of PKA substrates, even when the soluble adenylyl cyclase (sAC) was pharmacologically blocked [13]. Altogether, these results indicate that under acetylating conditions, both subunit dissociation and PKA activity can occur without an increase in cAMP concentrations, revealing lysine acetylation as a complementary mechanism for PKA modulation in sperm.

## DISCUSSION

The present study identifies lysine acetylation of the catalytic subunit of protein kinase A (PKA-C) as a novel post-translational modification that enhances enzymatic activity. By establishing an *in vitro* system in which recombinant PKA-C is acetylated by endogenous sperm acetyltransferases, combined with biochemical and spectroscopic approaches, we provide direct evidence that acetylation modulates PKA function through increased ATP affinity. In addition, increasing lysine acetylation in mouse sperm triggered the relocation of PKA subunits, mimicking capacitation, even in conditions where the soluble adenylyl cyclase is not activated. These findings reveal an additional regulatory layer for PKA activity during sperm capacitation and expand our understanding of how acetylation coordinates with canonical cAMP signaling to control sperm physiology.

One of the central findings of this work is that lysine acetylation selectively decreases *K*_m_ for ATP without significantly affecting the turnover rate (*V*_max_) for peptide substrates. NMR spectroscopy showed spectral perturbations in the glycine-rich loop and activation segment, structural elements directly involved in ATP coordination. Importantly, this work identified that Lys168 in PKA-C is acetylated, which is known to coordinate to ATP to facilitate phosphotransfer to the incoming substrate. Thus, this PTM may directly influence the electrostatic landscape of the active site, reducing repulsion of the negatively charged phosphate groups [42].

The observation that acetylation does not alter *K*_m_ for peptide substrates suggests that substrate recognition remains intact. Acetylation appears to fine-tunes the enzyme possibly by lowering the energetic threshold for ATP binding, effectively rendering PKA-C more catalytically efficient under limiting nucleotide concentrations. Recent evidence indicates that ATP levels decline during mouse sperm capacitation, reflecting the high energetic demands of motility and signaling cascades [43]. Early work demonstrated that ATP content decreases significantly as capacitation progresses and hyperactivation develops [44]. More recently, metabolic flux analyses have provided a mechanistic explanation for this phenomenon: capacitation promotes a shift in energy metabolism, with sperm increasing their reliance on glycolysis to sustain local ATP pools in the flagellum, while overall cellular ATP levels still decrease [45–47]. These findings highlight that ATP content declines during capacitation due to enhanced consumption by axonemal dyneins, ion transport, and kinase signaling. Thus, such a mechanism to increase affinity towards ATP may ensure sustained kinase activity even under metabolic stress. This process aligns with proteomic studies showing that sperm flagellar proteins, including kinases and metabolic enzymes, are extensively acetylated [18, 48], reinforcing the concept that acetylation could integrate metabolic states with signaling capacity.

*In vivo* acetylation of sperm proteins also promoted dissociation of PKA-C from its regulatory subunit (PKA-RII), mimicking capacitation-induced redistribution of PKA-RII [25]. In non-capacitated sperm, PKA-C and PKA-RII are tightly associated in a narrow axonemal compartment, consistent with an inactive state. Upon capacitation, PKA-RII relocates peripherally and associates with the CatSper channel complex in the plasma membrane, while PKA-C remains centrally positioned [25, 26]. Remarkably, acetylation induced a comparable spatial reorganization even in the absence of increased cAMP production, supporting a model in which lysine acetylation could decrease affinity over the regulatory–catalytic interface. Our data show that acetylation provides an alternative route to PKA activation in sperm, parallel to cAMP binding.

This dual regulation may explain a long-standing paradox in sperm signaling: despite transient increases in cAMP during early capacitation followed by a sustained decay to basal levels [10], PKA activity persists throughout the capacitation period. Acetylation of PKA-C could maintain kinase activity during later stages, once cAMP has declined, ensuring sustained phosphorylation of downstream effectors necessary for hyperactivation and acrosome reaction.

The discovery that acetylation promotes catalytic efficiency and *in vivo* dissociation of subunits highlights this PTM as a nexus between sperm metabolism and signal transduction. In sperm, metabolic plasticity is essential: ATP must be supplied locally to the flagellum for motility while simultaneously supporting signaling and membrane remodeling. By linking acetyl-CoA abundance to kinase activity, acetylation may provide a metabolic checkpoint that couples energy status to fertilization competence.

Several sperm glycolytic enzymes are acetylated [19], and lysine acetylation is known to modulate metabolic enzyme activity. Thus, acetylation may act as a global coordinator of both energy production and its utilization by kinases. For PKA, enhanced ATP affinity upon acetylation ensures that the phosphorylation of key substrates remains robust even under fluctuating ATP levels, while concomitant holoenzyme destabilization promotes persistent activation independent of cAMP synthesis. Together, these mechanisms would guarantee continuity of signaling required for capacitation.

Although phosphorylation remains the canonical regulatory mechanism for protein kinases, accumulating evidence indicates that acetylation also plays widespread roles in kinase biology. In hepatocytes, acetylation of Akt enhances membrane recruitment; in cancer cells, acetylation of MEK1/2 alters substrate specificity; and in yeast, acetylation of kinases modulates their stability and complex formation [49]. Our findings position PKA-C acetylation within this broader framework, demonstrating that it can modulate both catalytic activity and subcellular localization. Importantly, acetylation of lysines within catalytic loops has been described in other kinases, often altering ATP-binding affinity or catalytic efficiency [50–52]. The parallels with our findings on PKA-C suggest a conserved mechanism whereby acetylation fine-tunes kinase activity through electrostatic modulation of the active site. Given the evolutionary conservation of PKA, it is conceivable that acetylation serves as a generalizable regulatory principle across diverse cell types, extending beyond sperm biology.

The biological relevance of PKA acetylation in sperm extends to key functional outcomes. Capacitation requires tightly coordinated signaling events that culminate in hyperactivated motility and acrosome reaction [4]. Both processes rely on sustained PKA activity [8]. Acetylation provides a mechanism for temporal extension of PKA signaling beyond stimulation of the soluble adenylyl cyclase. On the other hand, misregulation of acetylation could contribute to male infertility: defects in deacetylase activity, acetyltransferase expression, or acetyl-CoA metabolism might impair sperm capacitation, leading to subfertility, and providing a mechanistic insight into how such alterations could compromise fertility by altering PKA signaling. Our findings reveal that lysine acetylation modulates the biological activity of PKA, expanding the known regulatory mechanisms of this kinase beyond classical paradigms. This represents a novel scope in both reproductive biology and broader cellular contexts, as it suggests that acetylation fine-tunes PKA activity independently of cAMP.

## Supplementary Material

Supplementary Files

This is a list of supplementary files associated with this preprint. Click to download.

- SUPPLEMENTALFIGURES.docx

## Figures and Tables

**Figure 1:**
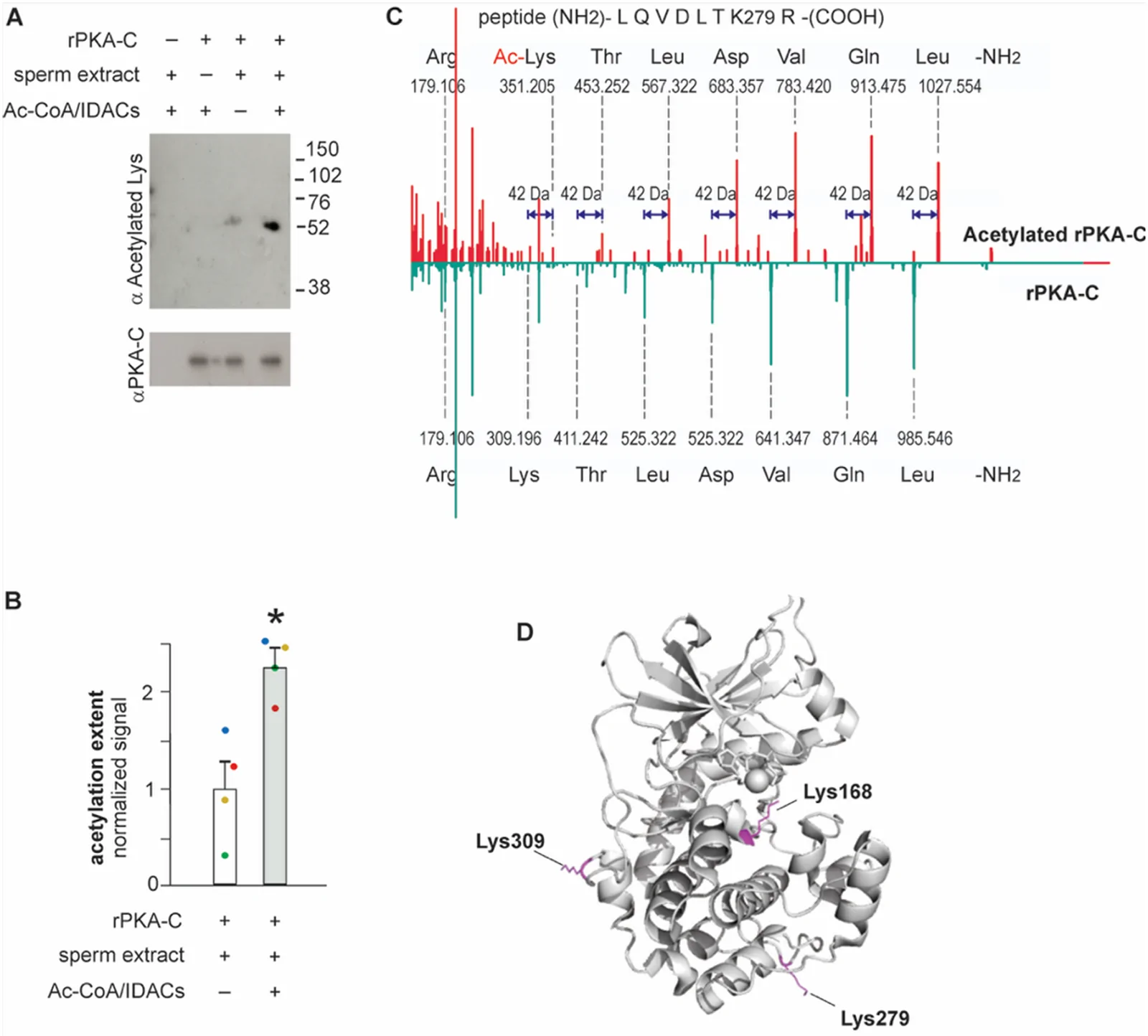
Recombinant PKA catalytic subunit is acetylated by sperm acetyltransferases. ***A,***Western blot analysis of rPKA-C fused to 6xHis-tag was subjected to *in vitro*acetylation using native acetyltransferases present in total sperm extracts, under conditions that either favored acetylation (Ac-CoA/iDACs) or not. The analysis used anti-Acetyl-Lys and anti-PKA-C antibodies. ***B***, Densitometry analysis of acetylated rPKA-C found after incubating the protein with sperm extracts in either the presence or absence of Ac-CoA/iDACs (mean ± SD, n=4, *p*<0.05). ***C,*** Three identified acetylated lysines are shown on a ribbon-diagram of a crystal PKA-C structure (PDB 3FJQ). ***D,*** LC-MS analysis of mass spectra for the peptide containing Lys279. The peptide was obtained after trypsin digestion of rPKA-C. The spectrum for the non-acetylated peptides is shown in green (lower panel) while the corresponding acetylated spectrum is shown in red (upper panel). In these spectra, the peptides are shown from carboxyterminal to aminoterminal. See spectra for the acetylated Lysines 168 and 309 in Supp Fig 1

**Figure 2:**
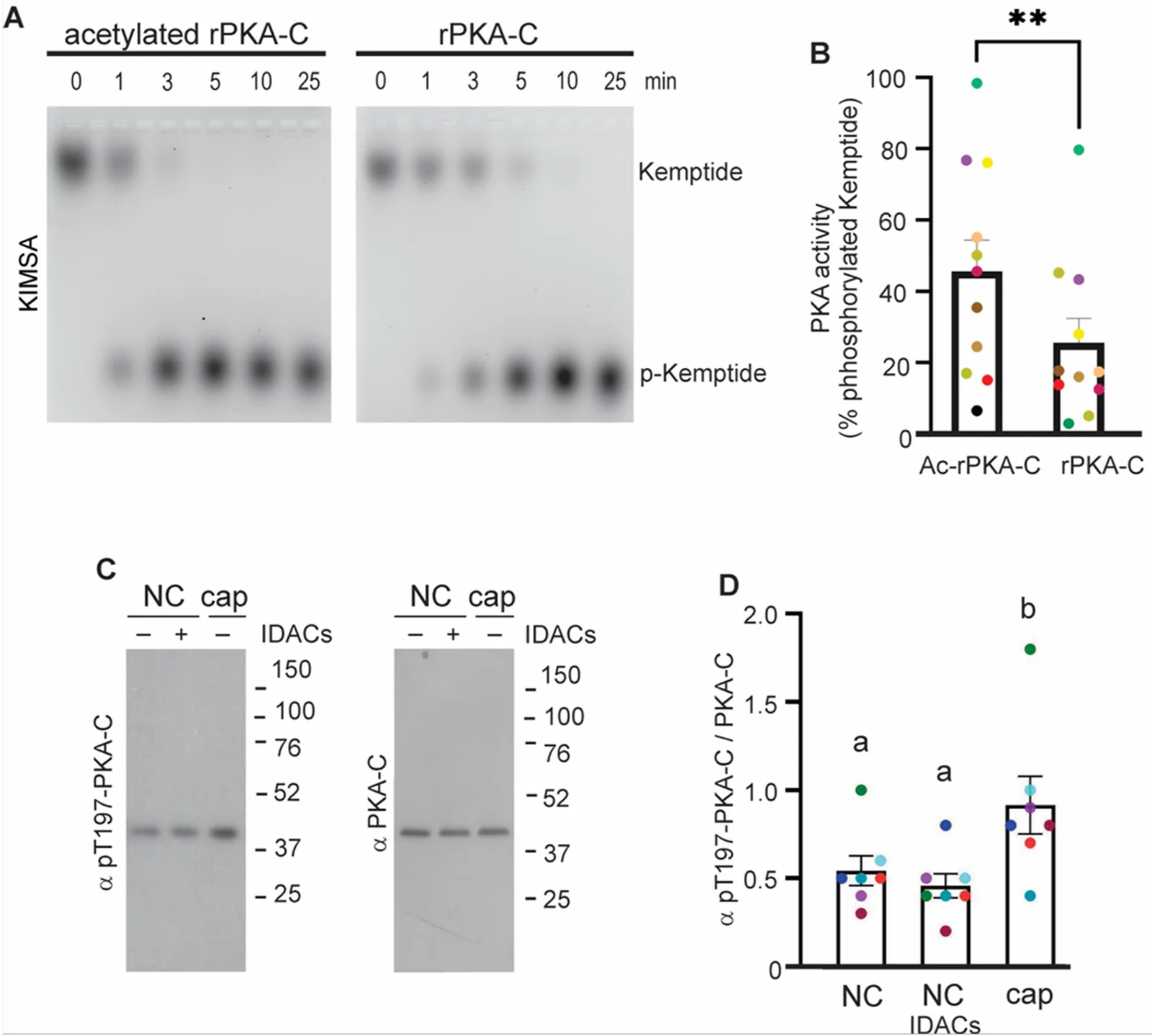
Acetylation enhances PKA catalytic activity without increasing phosphorylation on Thr197. ***A,*** Recombinant PKA-C was incubated with sperm extract under conditions that either favored acetylation (left panel) or not (right panel), and then recovered with Ni-beads before performing KiMSA assays. Different reaction times were analyzed. ***B,*** KiMSA with rPKA-C, either acetylated or not, were assessed at 5 min reaction period in KiMSA assays (mean ± SEM, n=11). Paired Student’s t-test was performed. **p<0.001***. C***, Sperm cells were incubated in NC conditions for 60 min in the presence or absence of IDACs or Cap conditions. Sperm extracts were then processed for SDS/PAGE and immunodetected by WB with anti-pThr197-PKA-C. A significant increase in phosphorylation on thr197-PKA-C was detected upon sperm capacitation, but IDACs addition to NC sperm did not increase phosphorylation. D, densitometry analysis of WB showed in C. One-way ANOVA followed by Tukey’s multiple comparisons test. Different letters indicate statistically significant differences (mean ± SEM; n = 7; p < 0.001).

**Figure 3:**
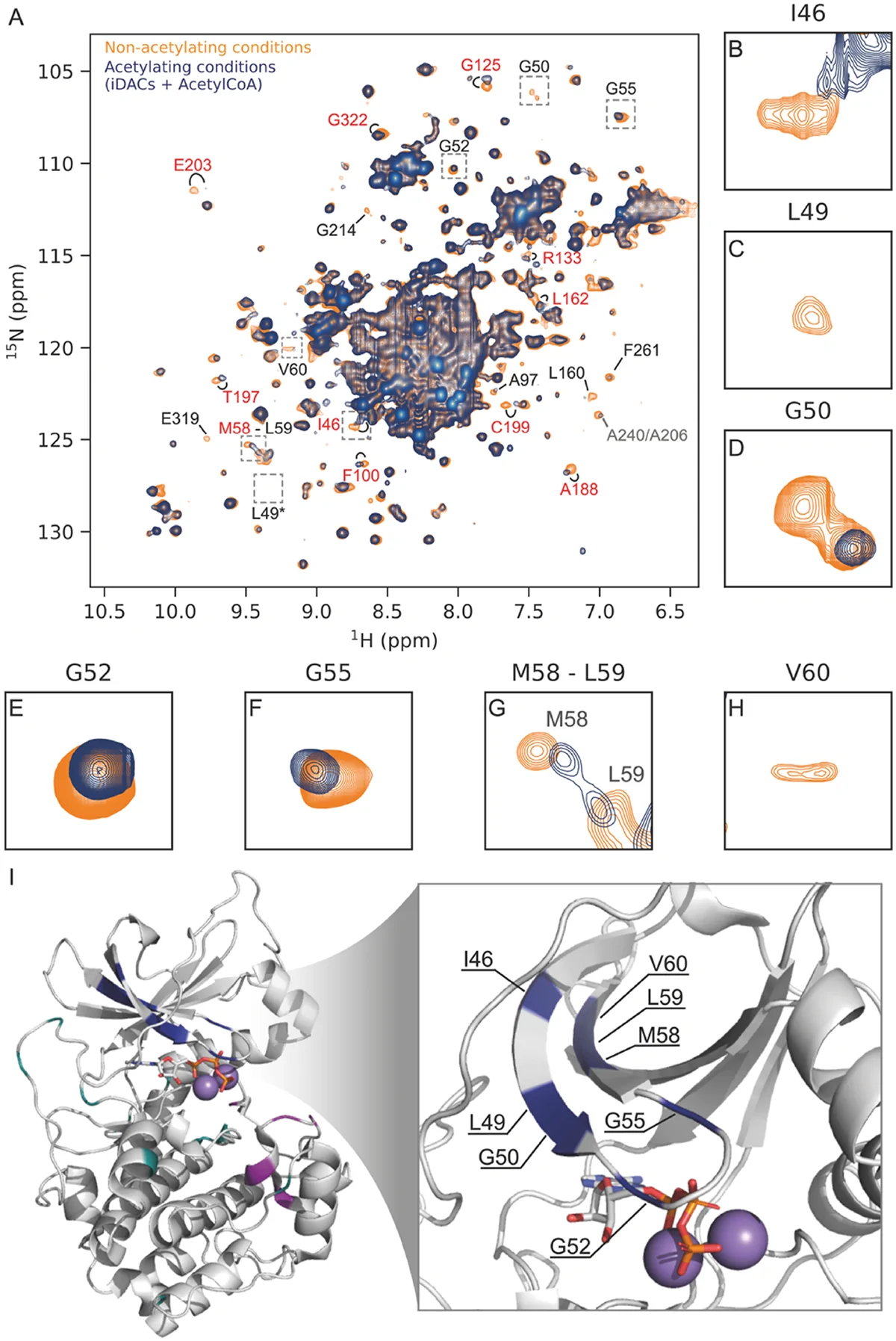
Acetylation induces changes in the ATP binding site of the catalytic subunit of PKA. ***A***, Overlay of ^1^H-^15^N HSQC NMR spectra of ^15^N-rPKA-C spiked in parallel sperm extracts in the absence (orange) and presence (blue) of acetyl-CoA and IDACs. Residues with chemical shift perturbations (CSP) above the threshold are labeled in red, while those with significant intensity reductions are labeled in black. Perturbed signals with overlapped assignments are shown in gray. Gray boxes highlight residues displayed in panels (B-H). ***B-H,*** Detailed views of spectral changes in the glycine-rich loop of PKA. ***I,*** Structural mapping of perturbed signals within the PKA structure (PDB 3FJQ). Residues exhibiting significant intensity changes or CSPs are color-coded by their structural location: glycine-rich loop (blue), activation segment (violet) or other regions (green). ATP and magnesium (II) are shown in stick and sphere representations, respectively.

**Figure 4:**
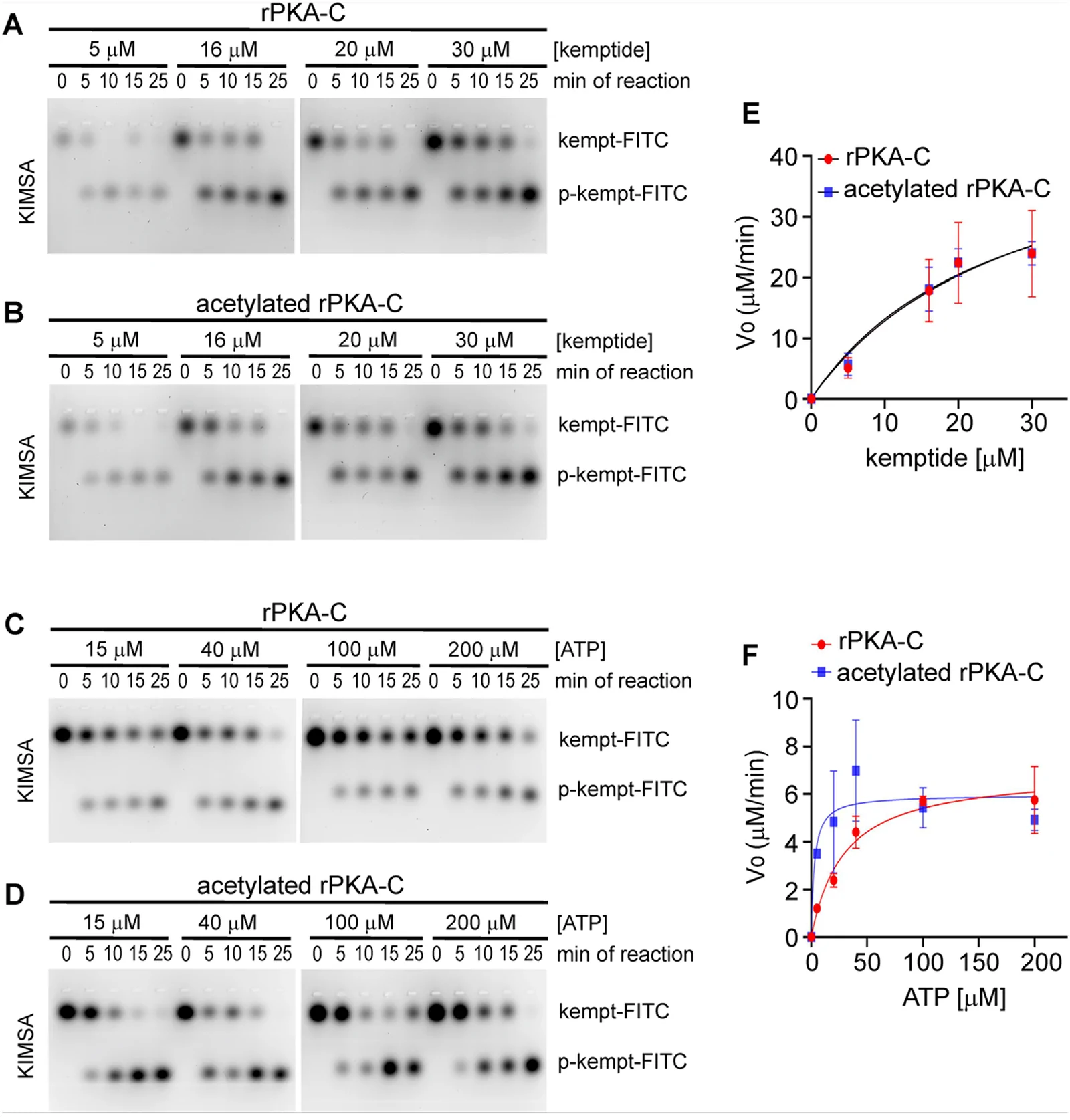
Lys-acetylation modifies the kinetic parameters for rPKA-C. Catalytic activity was measured using the KiMSA method for both the non-acetylated and the acetylated version of the protein. ***A and B***, KiMSA assays were performed using different amounts of kemptide over periods from 0 to 25 min at a fixed ATP concentration (200 μM) for either rPKA-C (***A***) or acetylated rPKA-C (***B***). ***C and D***, KiMSA assays were performed using diffrente amounts of ATP over periods from 0 to 25 min at a fixed Kemptide concentration (200 μM), for either rPKA-C (***C***) or acetylated rPKA-C (***D***). ***E,***Steady state kinetics of phosphoryl transfer were calculated for non-acetylated rPKA-C (red data points) and acetylated rPKA-C (blue data points) towards the peptide substrate Kemptide, following fitting with the Michaelis-Menten equation. ***F***, Steady state kinetics of phosphoryl transfer were calculated for non-acetylated rPKA-C (red data points) and acetylated rPKA-C (blue data points) towards ATP.

**Figure 5:**
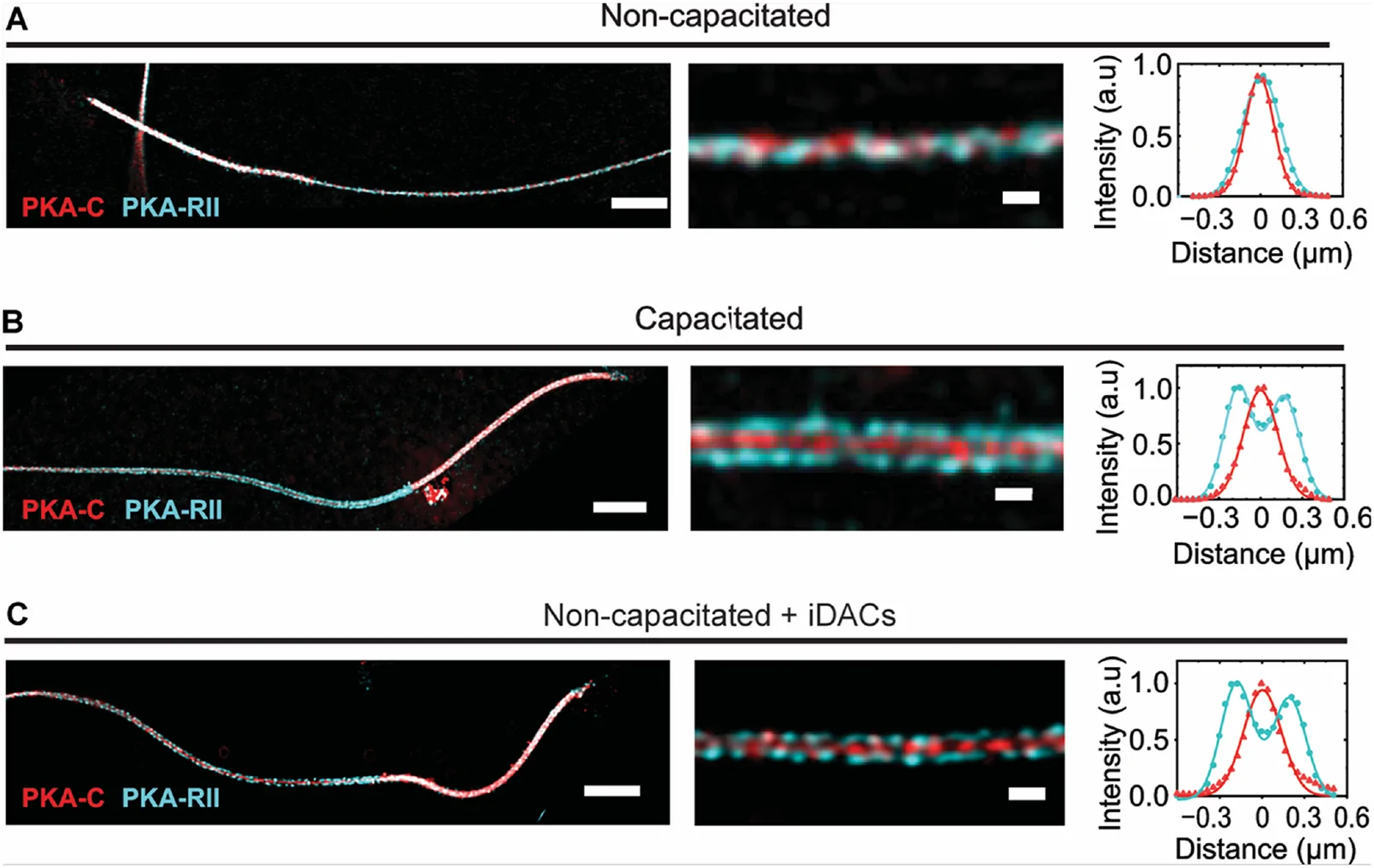
Pairwise two-color immunofluorescence imaging of PKA-C (red) and PKA-RII (cyan) in mouse sperm using LSM. In all images, the sperm head is oriented to the right, and the first fluorescent region of the flagellum on the right corresponds to the midpiece. In each section, the mid-panel shows a magnified view of the flagellum, and the right panel shows the intensity profile of the region shown in the mid panel across a plane that crosses the center of the flagellum. ***A,***Representative immunofluorescence of PKA-C and PKA-RII in a non-capacitated cell. The intensity cross-section exhibits a single-peak Gaussian-like profile for both labels. ***B,*** PKA-C and PKA-RII in a cell stimulated with capacitation-inducing medium. The intensity cross-section appears as a single peak for PKA-C and as a double-peak distribution for PKA-RII. ***C,*** PKA-C and PKA-RII in a cell incubated in non-capacitating conditions with deacetylase inhibitors (iDACs). The intensity cross-section appears as a single peak for PKA-C and as a double-peak distribution for PKA-RII. Scale bars for the upper and lower panels are 5 μm and 0.5 μm, respectively.

**Figure 6:**
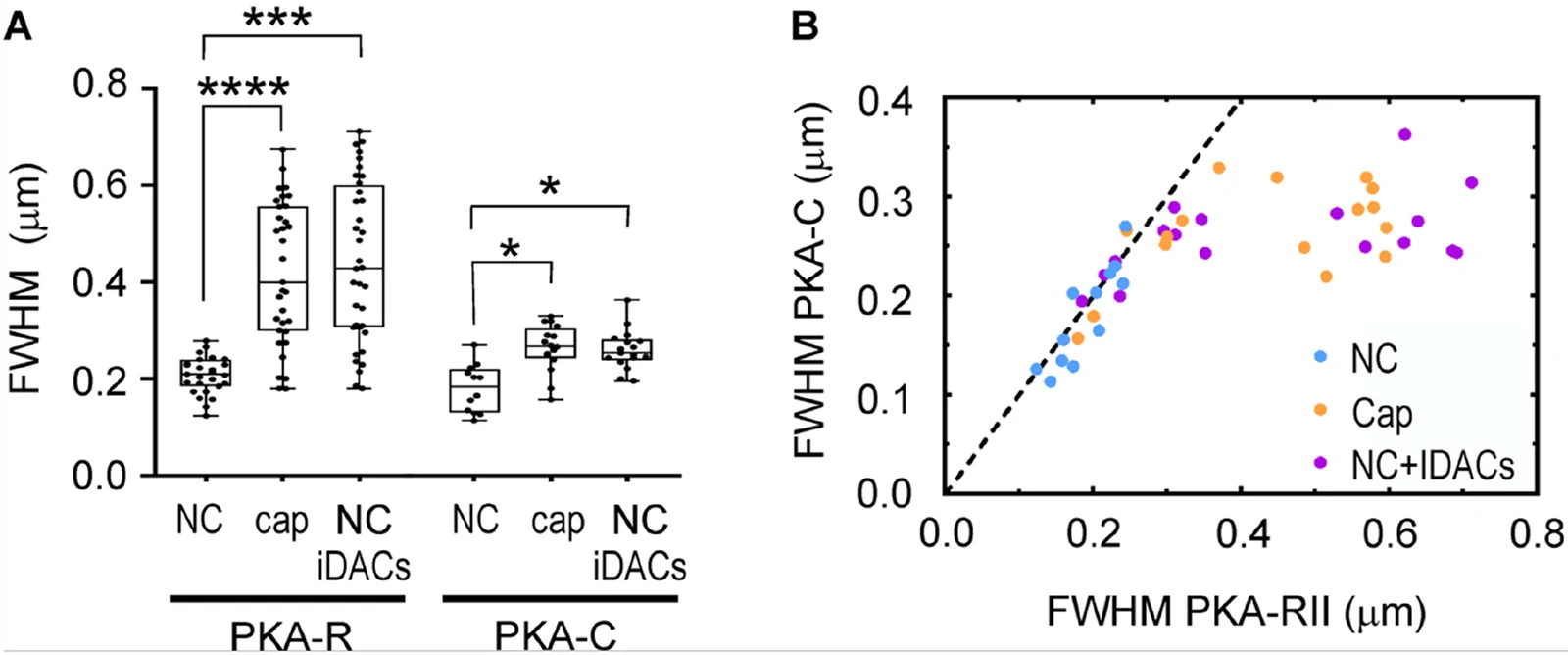
Quantification of the width of the distributions of PKA-RII and PKA-C, in the sperm principal piece. ***A*.** Box plots for the widths of PKA-RII and PKA-C in non-capacitated (NC) cells, cells incubated in capacitation media (cap), and cells NC media with deacetylase inhibitor (iDACs). The whiskers indicate the 5th and 95th percentiles. One-way ANOVA followed by Tukey’s multiple comparisons test (mean ± SD; *p < 0.05; *** p = 0.001 **** p < 0.001). ***B*.** Scatter plot of PKA-C width vs PKA-RII width in non-capacitating, capacitating, and non-capacitating + iDACs conditions. The dashed line indicates when both parameters are equal. A correlation is observed between the two widths in the cells incubated in non-capacitating medium and in a subset of the cells, both in capacitation-inducing medium and with iDACs. These subsets likely correspond to non-capacitated cells.

**Figure 7:**
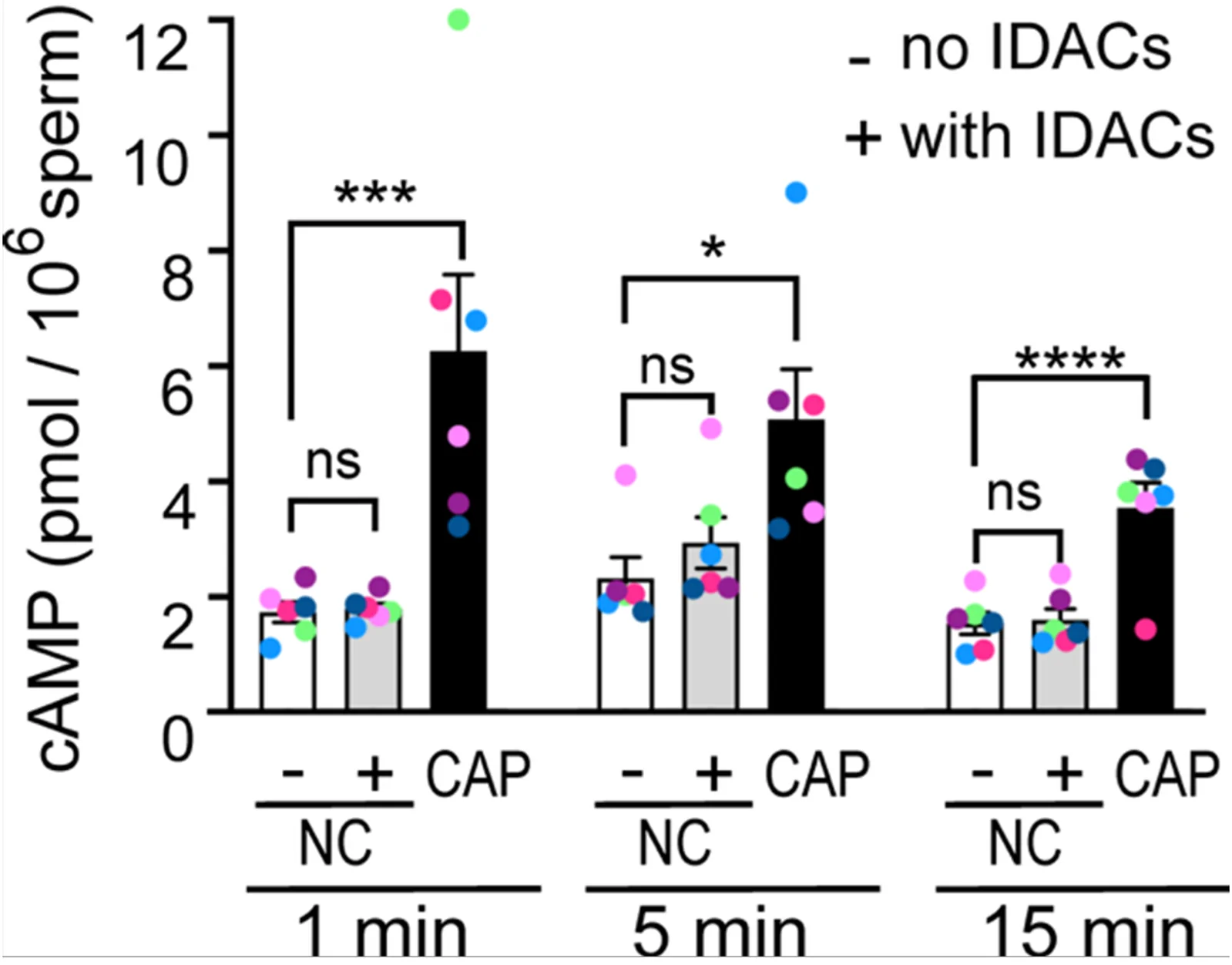
cAMP accumulated in sperm cells. cAMP accumulated in sperm cells. Cells were incubated for 1, 5, or 15 minutes in capacitating media, or in non-capacitating media containing or not iDACs. No cAMP increase was observed upon sperm incubations in the presence of iDACs. One-way ANOVA followed by Tukey’s multiple comparisons test (mean ± SEM; *p<0.05; *** p < 0.001; ****p< 0.0001).

**Tables 1.** Kinetic parameter for the acetylated and native PKA-C

	Ac-PKA-C	PKA-C	fold change
**Kemptide**			
*K*_m_ (μM)	25,08	27.98	−1.1
*V*_max_ (μM/min)	46.24	48.84	−1.05
**ATP**			
*K*_m_ (μM)	2.75	29.22	−10.62
*V*_max_ (μM/min)	5.96	6.97	−1.17

Kinetic parameter for Kemptide and ATP. The negative values of fold changes indicte a decrease in the value of the kinetic parameters for the acetylated PKA-C relative to the natibe PKA-C.

## Abbreviations

Ac-CoA: Acetyl-CoA
AGC: Automatic gain control
ATP: Adenosine triphosphate
BSA: Bovine serum albumin
cAMP: Cyclic adenosine monophosphate
CSP: Chemical shift perturbations
DTT: Dithiothreitol
EDTA: Ethylenediaminetetraacetic acid
FITC: Fluorescein isothiocyanate
FWHM: Full width at half maximum
HDACs: Deacetylases
IBMX: 3-Isobutyl-1-methylxanthine
iDACs: Deacetylase inhibitors
IPTG: Isopropyl-β-D-thiogalactopyranoside
KATs: Acetyltransferases
*K*cat: Catalytic constant
*K*m: Michaelis constant
LC-MS/MS: Liquid chromatography–mass spectrometry/mass spectrometry
LSM: Laser scanning microscopy
NAM: Nicotinamide
NGS: Normal goat serum
NMR: Nuclear magnetic resonance
PBS: Phosphate-buffered saline
PBS-T: Phosphate-buffered saline containing Tween-20
PFA: Paraformaldehyde
PhosSTOP: Phosphatase inhibitor cocktail
PKA: Protein kinase A
PKA-C: PKA catalytic subunit
PKA-R: PKA regulatory subunit
PTMs: Post-translational modifications
PVDF: Polyvinylidene fluoride membrane
rPKA-C: Recombinant mouse PKA catalytic subunit
RT: Room temperature
sAC: Soluble adenylyl cyclase
SD: Standard deviation
TBS-T: Tris-buffered saline containing Tween-20
TSA: Trichostatin A
*V*max: Maximum velocity

## References

[1] AUSTIN CR. The ‘Capacitation’ of the Mammalian Sperm. Volume 170. Nature Publishing Group; 1952. pp. 326–326. [cited 2025 Mar 6];. 10.1038/170326a0. Nature 1952 170:4321 [Internet]. 12993150

[2] Chang MC. Fertilizing capacity of spermatozoa deposited into the fallopian tubes. Nature. 1951;168:697–8. 10.1038/168697b0 14882325

[3] Toyoda Y, Yokoyama M, Hosi T. Studies on the fertilization of mouse eggs in vitro. Japanese J Anim Reprod Japanese Soc Anim Reprod. 1971;16:152–7. 10.1262/jrd1955.16.152.

[4] Stival C, Puga Molina LCLDC, Paudel B, Buffone MGMG, Visconti PEPE, Krapf D. Sperm capacitation and acrosome reaction in mammalian sperm [Internet]. Adv. Anat. Embryol. Cell Biol. Springer Verlag; 2016 [cited 2023 Oct 25]. 10.1007/978-3-319-30567-7_5 27194351

[5] Jaiswal BS, Conti M. Calcium regulation of the soluble adenylyl cyclase expressed in mammalian spermatozoa. Proc Natl Acad Sci U S A [Internet]. National Academy of Sciences; 2003 [cited 2026 Jul 24];100:10676–81. 10.1073/PNAS.1831008100;WGROUP:STRING:PUBLICATION. PMC196863 12958208

[6] Luque GM, Jabloñski M, Schiavi-Ehrenhaus LJ, del Prado RC, Balbach M, Romarowski A, Bovine serum albumin-induced calcium influx triggers soluble adenylyl cyclase activation and cyclic AMP signalling pathways in mouse sperm capacitation. J Physiol [Internet] J Physiol. 2025;603:2633–53. 10.1113/JP288389. [cited 2026 Jun 26]. PMC12856760 40320899

[7] Knighton DR, Zheng J, Ten Eyck LF, Ashford VA, Xuong NH, Taylor SS, Crystal structure of the catalytic subunit of cyclic adenosine monophosphate-dependent protein kinase. Sci [Internet] Sci. 1991;253:407–14. 10.1126/science.1862342. [cited 2026 Mar 29];. 1862342

[8] Baro Graf C, Ritagliati C, Stival C, Luque GM, Gentile I, Buffone MG Everything you ever wanted to know about PKA regulation and its involvement in mammalian sperm capacitation. Mol Cell Endocrinol [Internet]. Elsevier Ireland Ltd; 2020 [cited 2021 Mar 23];518:110992. 10.1016/j.mce.2020.110992 32853743

[9] Leung MR, Sun C, Zeng J, Anderson JR, Niu Q, Huang W Structural diversity of axonemes across mammalian motile cilia. Nature 2025 637:8048 [Internet]. Nature Publishing Group; 2025 [cited 2025 Jun 16];637:1170–7. 10.1038/s41586-024-08337-5 PMC11779644 39743588

[10] Balbach M, Ghanem L, Rossetti T, Kaur N, Ritagliati C, Ferreira J, Soluble adenylyl cyclase inhibition prevents human sperm functions essential for fertilization. Mol Hum Reprod [Internet]. 2021;27:1–13. 10.1093/molehr/gaab054. [cited 2023 Oct 25];. PMC8473925 34463764

[11] Grunewald S, Paasch U, Glander HJ, Anderegg U. Mature human spermatozoa do not transcribe novel RNA. Andrologia [Internet]. Wiley, Ltd; 2005 [cited 2026 Apr 15];37:69–71. 10.1111/J.1439-0272.2005.00656.X;REQUESTEDJOURNAL:JOURNAL:14390272;WGROUP:STRING:PUBLICATION. 16026427

[12] Iniesta-Cuerda M, Valentova I, Moravec J, Liška F, Králíčková M, Krapf D SIRT1 haploinsufficiency recapitulates age-associated subfertility through sperm α-tubulin hyperacetylation. Biology Direct. 2026 21:1 [Internet]. BioMed Central; 2026 [cited 2026 Apr 15];21:45-. 10.1186/S13062-025-00710-2 PMC13067630 41882697

[13] Ritagliati C, Luque GM, Stival C, Baro Graf C, Buffone MG, Krapf D. Lysine acetylation modulates mouse sperm capacitation. Sci Rep [Internet] Springer US. 2018;8:1–14. 10.1038/s41598-018-31557-5. [cited 2021 Mar 23];. PMC6127136 30190490

[14] Wang Q, Zhang Y, Yang C, Xiong H, Lin Y, Yao J, Acetylation of metabolic enzymes coordinates carbon source utilization and metabolic flux. Sci [Internet] Sci. 2010;327:1004–7. 10.1126/SCIENCE.1179687. [cited 2026 Jul 24];. PMC4183141 20167787

[15] Zhao S, Xu W, Jiang W, Yu W, Lin Y, Zhang T, Regulation of cellular metabolism by protein lysine acetylation. Sci [Internet] Sci. 2010;327:1000–4. 10.1126/SCIENCE.1179689. [cited 2026 Jul 24];. PMC3232675 20167786

[16] Kim SC, Sprung R, Chen Y, Xu Y, Ball H, Pei J, Substrate and Functional Diversity of Lysine Acetylation Revealed by a Proteomics Survey. Mol Cell [Internet] Mol Cell. 2006;23:607–18. 10.1016/j.molcel.2006.06.026. [cited 2026 Jul 24];. 16916647

[17] Gu W, Roeder RG. Activation of p53 sequence-specific DNA binding by acetylation of the p53 C-terminal domain. Cell [Internet]. Volume 90. Cell; 1997. pp. 595–606. [cited 2026 Jul 24];. 10.1016/S0092-8674(00)80521-8. 9288740

[18] Choudhary C, Kumar C, Gnad F, Nielsen ML, Rehman M, Walther TC, Lysine acetylation targets protein complexes and co-regulates major cellular functions. Sci [Internet] Sci. 2009;325:834–40. 10.1126/SCIENCE.1175371. [cited 2026 Jul 24];. 19608861

[19] Sun G, Jiang M, Zhou T, Guo Y, Cui Y, Guo X, Insights into the lysine acetylproteome of human sperm. J Proteom Elsevier. 2014;109:199–211. 10.1016/j.jprot.2014.07.002. 25038526

[20] Tian Y, Wang H, Pan T, Hu X, Ding J, Chen Y, Global proteomic analyses of lysine acetylation, malonylation, succinylation, and crotonylation in human sperm reveal their involvement in male fertility. J Proteom [Internet] Elsevier B V. 2024. 10.1016/j.jprot.2024.105213. [cited 2026 Apr 15];303. 38797435

[21] Yu H, Diao H, Wang C, Lin Y, Yu F, Lu H, Acetylproteomic Analysis Reveals Functional Implications of Lysine Acetylation in Human Spermatozoa (sperm). Mol Cell Proteomics [Internet]. Volume 14. American Society for Biochemistry and Molecular Biology Inc.; 2015. p. 1009. [cited 2026 Apr 15];. 10.1074/MCP.M114.041384. PMC4390248 25680958

[22] Narita T, Weinert BT, Choudhary C. Functions and mechanisms of non-histone protein acetylation. Nat Rev Mol Cell Biol [Internet] Nat Rev Mol Cell Biol. 2019;20:156–74. 10.1038/s41580-018-0081-3. [cited 2026 Mar 29];. 30467427

[23] Taylor SS, Ilouz R, Zhang P, Kornev AP. Assembly of allosteric macromolecular switches: lessons from PKA. Nat Rev Mol Cell Biol [Internet] Nat Rev Mol Cell Biol. 2012;13:646–58. 10.1038/NRM3432. [cited 2026 Apr 15];. PMC3985763 22992589

[24] Choudhary C, Weinert BT, Nishida Y, Verdin E, Mann M. The growing landscape of lysine acetylation links metabolism and cell signalling. Nat Rev Mol Cell Biol [Internet] Nat Rev Mol Cell Biol. 2014;15:536–50. 10.1038/nrm3841. [cited 2026 Mar 29];. 25053359

[25] Novero AG, Matamoros-Volante A, Gomez-Olivieri LR, Stival C, Luque GM, Szalai AM, Dynamic relocation of PKA regulatory subunits during sperm capacitation: Linking PKA to the CatSper signaling complex. Volume 122. National Academy of Sciences; 2025. p. e2501741122. [cited 2025 Aug 11];. 10.1073/PNAS.2501741122/SUPPL_FILE/PNAS.2501741122.SAPP.PDF. Proc Natl Acad Sci U S A [Internet]. PMC12167957 40478882

[26] Stival C, Ritagliati C, Xu X, Gervasi MGMG, Luque GMGM, Graf CBCB, Disruption of protein kinase A localization induces acrosomal exocytosis in capacitated mouse sperm. J Biol Chem Am Soc Biochem Mol Biology Inc. 2018;293:9435–47. 10.1074/jbc.RA118.002286. PMC6005427 29700114

[27] Narayana N, Cox S, Shaltiel S, Taylor SS, Xuong N-H. Crystal Structure of a Polyhistidine-Tagged Recombinant Catalytic Subunit of cAMP-Dependent Protein Kinase Complexed with the Peptide Inhibitor PKI(5–24) and Adenosine †. 1997 [cited 2026 Apr 15]; 10.1021/BI961947 9109651

[28] Yonemoto W, McGlone ML, Grant B, Taylor SS. Autophosphorylation of the catalytic subunit of cAMP-dependent protein kinase in Escherichia coli. Protein Eng [Internet]. Oxford Academic; 1997 [cited 2026 Mar 29];10:915–25. 10.1093/protein/10.8.915 9415441

[29] Wu Z, Jin Y, Chen B, Gugger MK, Wilkinson-Johnson CL, Tiambeng TN, Comprehensive Characterization of Recombinant Catalytic Subunit of cAMP-Dependent Protein Kinase by Top-Down Mass Spectrometry. Volume 30. Springer; 2019. p. 2561. [cited 2026 Apr 15];. 10.1007/S13361-019-02341-0. J Am Soc Mass Spectrom [Internet]. PMC6922056 31792770

[30] Martín-Bernabé A, Tarragó-Celada J, Cunin V, Michelland S, Cortés R, Poignant J, Quantitative Proteomic Approach Reveals Altered Metabolic Pathways in Response to the Inhibition of Lysine Deacetylases in A549 Cells under Normoxia and Hypoxia. Int J Mol Sci [Internet] Int J Mol Sci. 2021. 10.3390/IJMS22073378. [cited 2026 Apr 15];22. PMC8036653 33806075

[31] Novero AG, Steeman TJ, Curcio C, Buccolini L, Binolfi A, Krapf D Kinase Mobility Shift Assay (KiMSA) for Assessing Protein Kinase A Activity. Bio Protoc [Internet]. Bio Protoc; 2025 [cited 2026 Mar 29];15. 10.21769/BioProtoc.5366 PMC12245630 40655420

[32] Novero AG, Curcio C, Steeman TJ, Binolfi A, Krapf D, Buffone MG, A versatile kinase mobility shift assay (KiMSA) for PKA analysis and cyclic AMP detection in sperm physiology (and beyond). Front Cell Dev Biol [Internet] Front Media SA. 2024;12:1356566. 10.3389/fcell.2024.1356566. [cited 2026 Mar 29];. PMC10912184 38444827

[33] Link AJ, LaBaer J. In-gel trypsin digest of gel-fractionated proteins. Cold Spring Harb Protoc. 2009. 10.1101/PDB.PROT5110. [cited 2026 Jul 24];2009. Cold Spring Harb Protoc [Internet]. 20147065

[34] Perez-Riverol Y, Bandla C, Kundu DJ, Kamatchinathan S, Bai J, Hewapathirana S The PRIDE database at 20 years: 2025 update. Nucleic Acids Res [Internet]. Nucleic Acids Res; 2025 [cited 2026 Jul 24];53:D543–53. 10.1093/NAR/GKAE1011 PMC11701690 39494541

[35] Pervushin K, Vögeli B, Eletsky A. Longitudinal (1)H relaxation optimization in TROSY NMR spectroscopy. J Am Chem Soc [Internet] J Am Chem Soc. 2002;124:12898–902. 10.1021/ja027149q. [cited 2026 Mar 29];. 12392438

[36] Skinner SP, Fogh RH, Boucher W, Ragan TJ, Mureddu LG, Vuister GW. CcpNmr AnalysisAssign: a flexible platform for integrated NMR analysis. Journal of Biomolecular NMR 2016 66:2 [Internet]. Springer; 2016 [cited 2026 Mar 29];66:111–24. 10.1007/s10858-016-0060-y PMC5095159 27663422

[37] Walker C, Wang Y, Olivieri C, Karamafrooz A, Casby J, Bathon K, Cushing’s syndrome driver mutation disrupts protein kinase A allosteric network, altering both regulation and substrate specificity. Sci Adv [Internet] Sci Adv. 2019. 10.1126/SCIADV.AAW9298. [cited 2026 Apr 15];5. PMC6713507 31489371

[38] Helmus JJ, Jaroniec CP. Nmrglue: an open source Python package for the analysis of multidimensional NMR data. Journal of Biomolecular NMR 2013 55:4 [Internet]. Springer; 2013 [cited 2026 Mar 29];55:355–67. 10.1007/s10858-013-9718-x PMC3636164 23456039

[39] Johnson DA, Akamine P, Radzio-Andzelm E, Madhusudan, Taylor SS. Dynamics of cAMP-Dependent Protein Kinase. Chem Rev [Internet]. Volume 101. American Chemical Society; 2001. pp. 2243–70. [cited 2026 Apr 15];. 10.1021/CR000226K. 11749372

[40] Steichen JM, Iyer GH, Li S, Saldanha A, Deal MS, Woods VL, Global Consequences of Activation Loop Phosphorylation on Protein Kinase A. J Biol Chem [Internet]. 2009;285:3825. 10.1074/JBC.M109.061820. [cited 2026 Apr 15];. PMC2823524 19965870

[41] Dose A, Liokatis S, Theillet FX, Selenko P, Schwarzer D. NMR Profiling of Histone Deacetylase and Acetyl-transferase Activities in Real Time. ACS Chem Biol [Internet]. Volume 6. American Chemical Society; 2011. pp. 419–24. [cited 2026 Mar 29];. 10.1021/cb1003866. 21302972

[42] Meharena HS, Fan X, Ahuja LG, Keshwani MM, McClendon CL, Chen AM Decoding the Interactions Regulating the Active State Mechanics of Eukaryotic Protein Kinases. PLoS Biol [Internet]. PLoS Biol; 2016 [cited 2026 Mar 29];14. 10.1371/journal.pbio.2000127 PMC5130182 27902690

[43] Balbach M, Ghanem L, Violante S, Kyaw A, Romarowski A, Cross JR, Capacitation induces changes in metabolic pathways supporting motility of epididymal and ejaculated sperm. Front Cell Dev Biol Front Media SA. 2023;11:1160154. 10.3389/fcell.2023.1160154. PMC10335746 37440924

[44] Mukai C, Okuno M. Glycolysis plays a major role for adenosine triphosphate supplementation in mouse sperm flagellar movement. Biol Reprod [Internet] Biol Reprod. 2004;71:540–7. 10.1095/biolreprod.103.026054. [cited 2026 Mar 29];. 15084484

[45] Tourmente M, Roldan ERS. Mass-Specific Metabolic Rate Influences Sperm Performance through Energy Production in Mammals. PLoS One [Internet] Public Libr Sci. 2015;10:e0138185. 10.1371/journal.pone.0138185. [cited 2026 Mar 29];. PMC4570794 26371474

[46] Tourmente M, Sansegundo E, Rial E, Roldan ERS. Capacitation promotes a shift in energy metabolism in murine sperm. Front Cell Dev Biol [Internet] Front Media S A. 2022. 10.3389/FCELL.2022.950979/PDF. [cited 2025 Jun 13];10. PMC9445201 36081906

[47] Tourmente M, Sansegundo E, Rial E, Roldan ERS. Bioenergetic changes in response to sperm capacitation and two-way metabolic compensation in a new murine model. Volume 80. Springer; 2022. pp. 11–. [cited 2026 Mar 29];. 10.1007/s00018-022-04652-0. Cellular and Molecular Life Sciences 2022 80:1 [Internet]. PMC9763147 36534181

[48] Castillo J, Amaral A, Oliva R. Sperm nuclear proteome and its epigenetic potential. Andrology [Internet]. Volume 2. Blackwell Publishing Ltd; 2014. pp. 326–38. [cited 2026 Mar 29];. 10.1111/j.2047-2927.2013.00170.x. 24327354

[49] Zhang J, Sprung R, Pei J, Tan X, Kim S, Zhu H Lysine acetylation is a highly abundant and evolutionarily conserved modification in Escherichia coli. Molecular and Cellular Proteomics [Internet]. Mol Cell Proteomics; 2009 [cited 2026 Mar 29];8:215–25. 10.1074/mcp.M800187-MCP200 PMC2634580 18723842

[50] Shang S, Liu J, Hua F. Protein acylation: mechanisms, biological functions and therapeutic targets. Volume 7. Nature Publishing Group; 2022. p. 396. [cited 2026 Mar 29];. 10.1038/s41392-022-01245-y. Signal Transduction and Targeted Therapy 2022 7:1 [Internet]. PMC9797573 36577755

[51] Lee J, Ko YU, Chung Y, Yun N, Kim M, Kim K The acetylation of cyclin-dependent kinase 5 at lysine 33 regulates kinase activity and neurite length in hippocampal neurons. Scientific Reports 2018 8:1 [Internet]. Nature Publishing Group; 2018 [cited 2026 Mar 29];8:13676-. 10.1038/s41598-018-31785-9 PMC6135752 30209341

[52] Trosky JE, Li Y, Mukherjee S, Keitany G, Ball H, Orth K. VopA inhibits ATP binding by acetylating the catalytic loop of MAPK kinases. J Biol Chem [Internet] J Biol Chem. 2007;282:34299–305. 10.1074/jbc.M706970200. [cited 2026 Mar 29];. 17881352

